# Supplementary figures and images for: L444P Gba1 mutation increases formation and spread of α-synuclein deposits in mice injected with mouse α-synuclein pre-formed fibrils
Source: PLoS One. 2020 Aug 24;15(8):e0238075. doi: 10.1371/journal.pone.0238075 (PMC7444808; doi:10.1371/journal.pone.0238075)

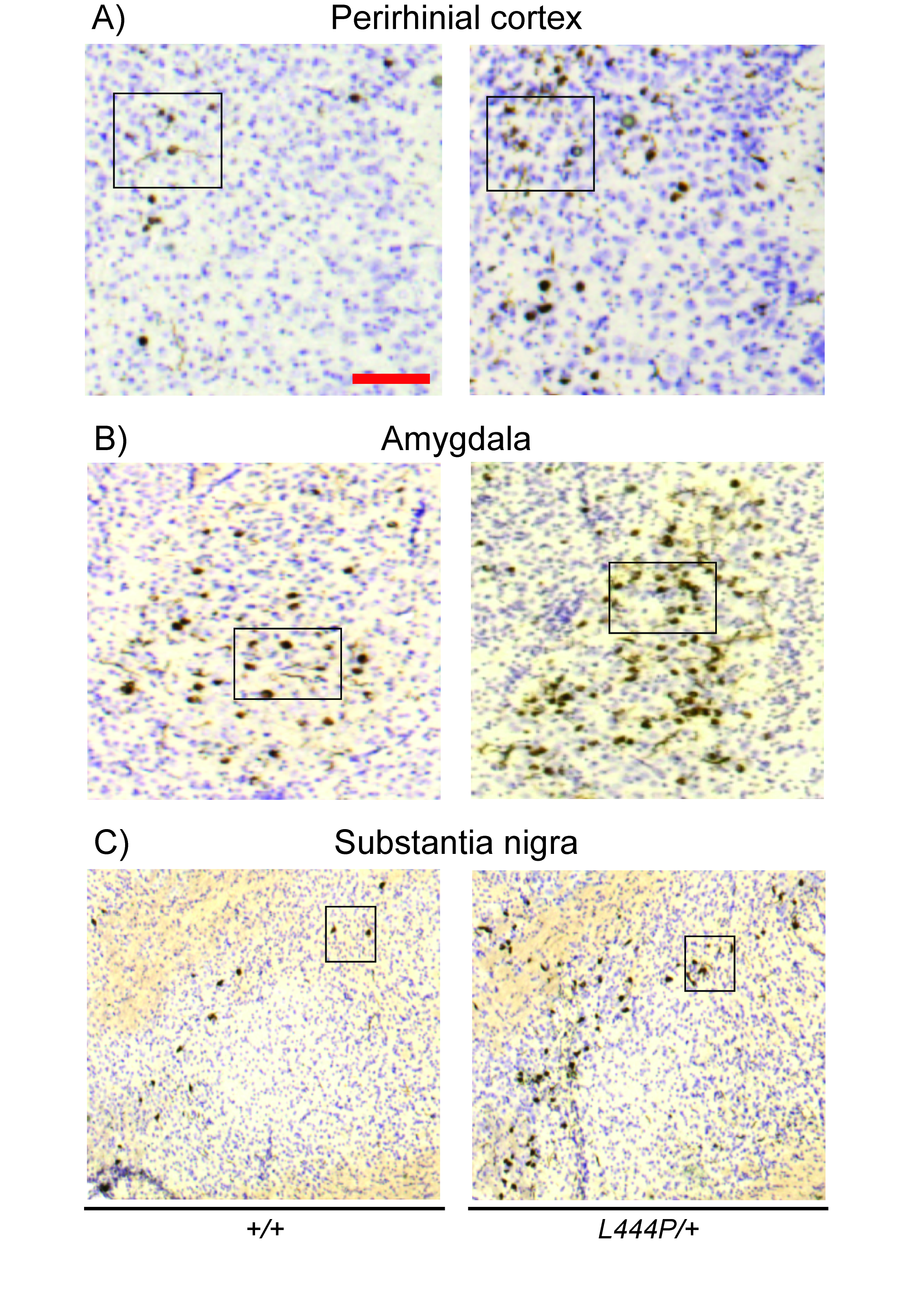

Supplement: S1 Fig — (A) Increased p-αSYN pathology in the perirhinal cortex at the level of 2.2mm posterior to the injection site (-2.0mm from the bregma) in the ipsilateral hemisphere of L444P/+ mice compared to their wild-type control littermates. (B) Increased p-αSYN pathology in the lateral amygdaloid nuclei at the level of 2.2mm posterior to the injection site (-2.0mm from the bregma) in the ipsilateral hemisphere of L444P/+ mice compared to their wild-type control littermates. (C) Increased p-αSYN pathology in substantia nigra pars compacta at the level of 3.7mm posterior to the injection site (-3.5mm from the bregma) in the ipsilateral hemisphere of L444P/+ mice compared to their wild-type control littermates. Scale bars = 100µm. Representative images shown. In total ten +/+ and four L444P/+ mice were analyzed. (TIF) [file pone.0238075.s001.tif]

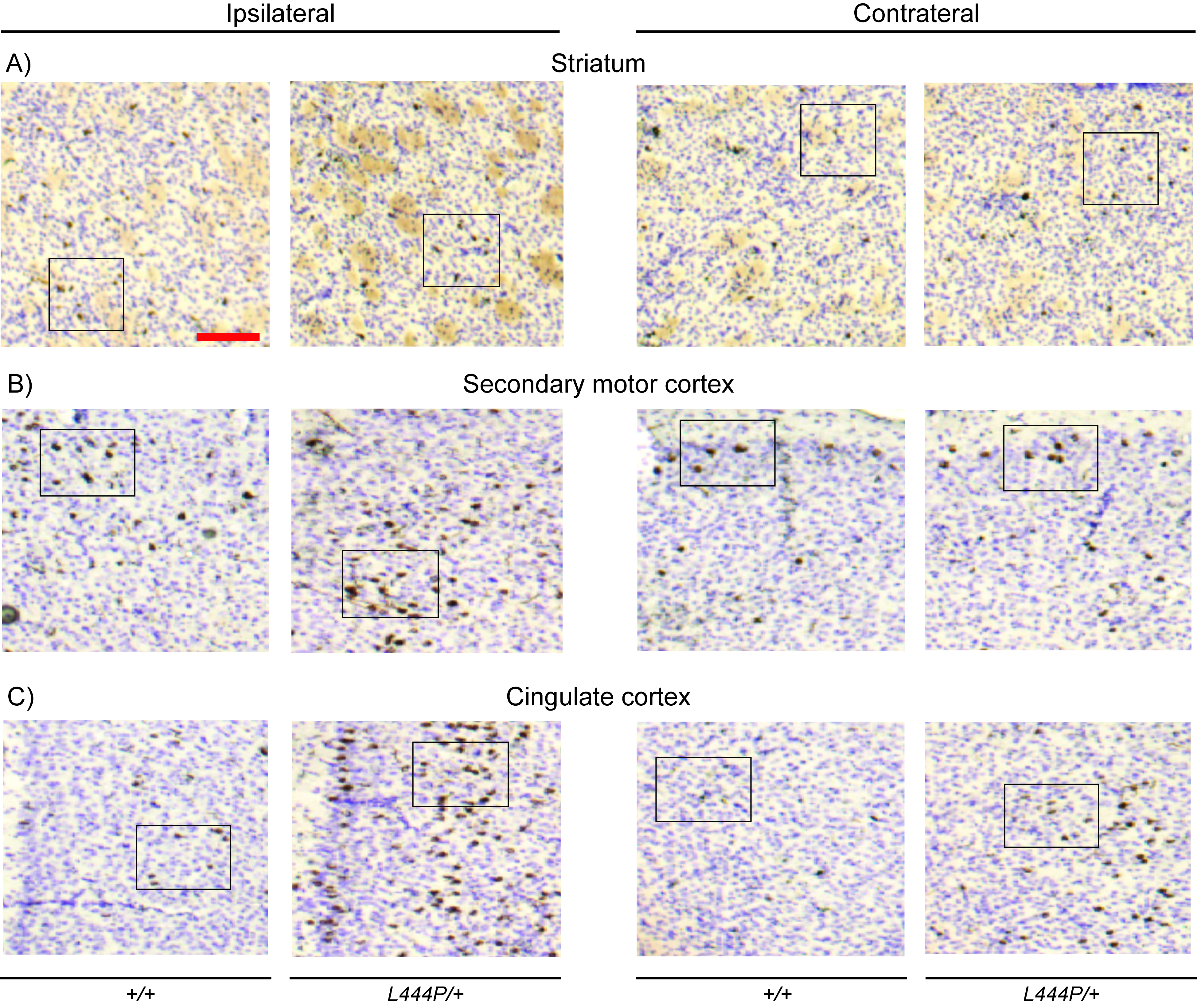

Supplement: S2 Fig — (A) Increased p-αSYN pathology in the striatal tissue at the level of 0.6mm anterior to the injection site (+0.8mm from the bregma) in the ipsilateral and contralateral hemispheres of L444P/+ mice compared to their wild-type control littermates. (B) Increased p-αSYN pathology in the cortical tissue at the level of 0.6mm anterior to the injection site (+0.8mm from the bregma) in layer 5 of the secondary motor cortex in the ipsilateral and in layer 2 of the secondary motor cortex in the contralateral hemisphere of L444P/+ mice compared to their wild-type control littermates. (C) Increased p-αSYN pathology in the cortical tissue at the level of 0.6mm anterior to the injection site (+0.8mm from the bregma) in the cingulate cortex in the ipsilateral and contralateral hemispheres of L444P/+ mice compared to their wild-type control littermates. Scale bars = 100µm. Representative images shown. Ten +/+ and four L444P/+ mice were analyzed in total. (TIF) [file pone.0238075.s002.tif]

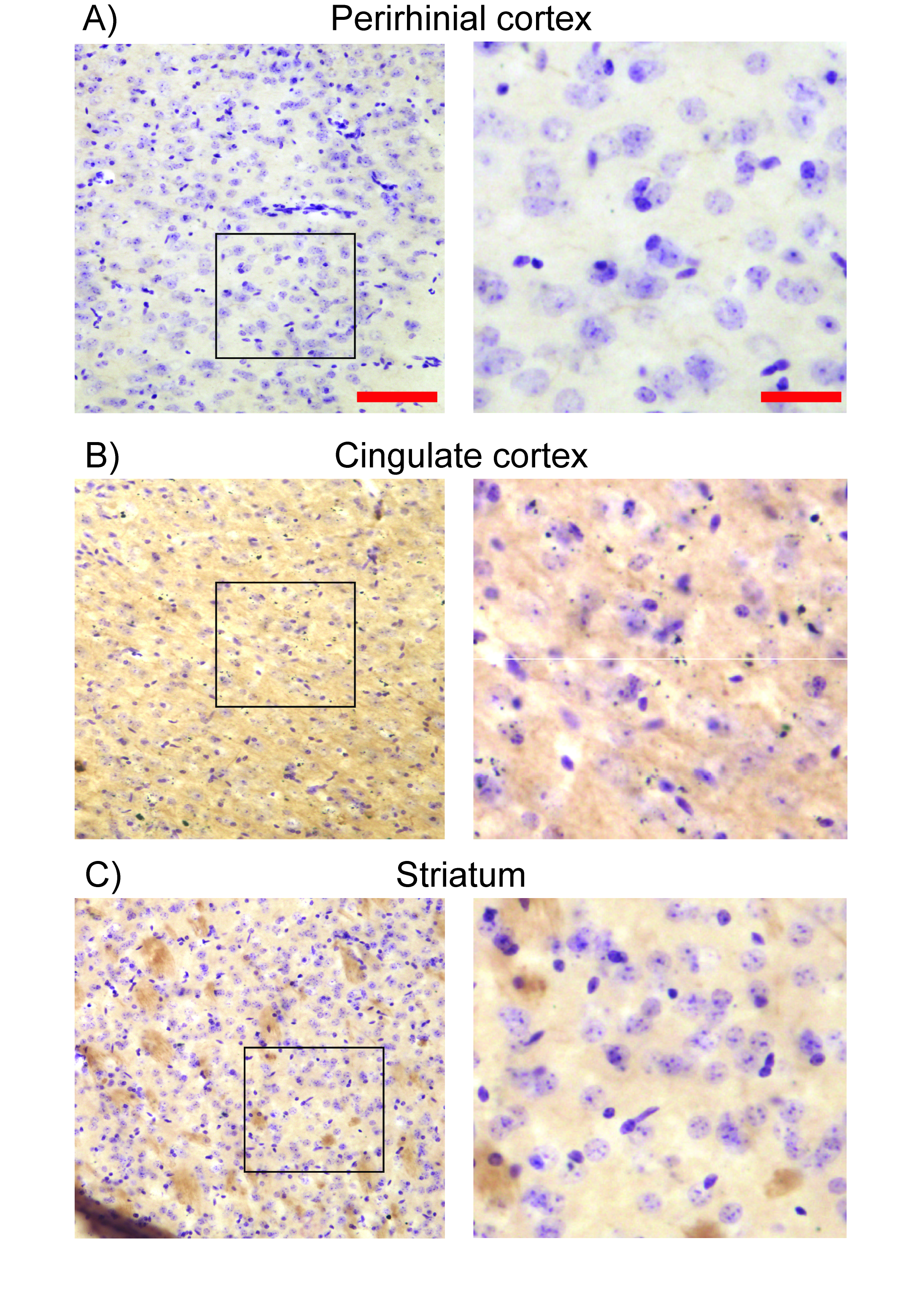

Supplement: S3 Fig — (A) No p-αSYN pathology in the perirhinal cortex at the level of 2.2mm posterior to the injection site (-2.0mm from the bregma) in the ipsilateral hemisphere of wild-type controls. (B) No p-αSYN pathology in the cortical tissue at the level of 0.6mm anterior to the injection site (+0.8mm from the bregma) in the cingulate cortex in the ipsilateral hemisphere of wild-type controls. (C) No p-αSYN pathology in the striatal tissue at the level of 0.6mm anterior to the injection site (+0.8mm from the bregma) in the ipsilateral hemispheres of wild-type controls. (B) Scale bars at low magnification = 100µm. Scale bars at high magnification = 25µm. Representative images shown. Ten wild-type mice were analyzed in total. (TIF) [file pone.0238075.s003.tif]

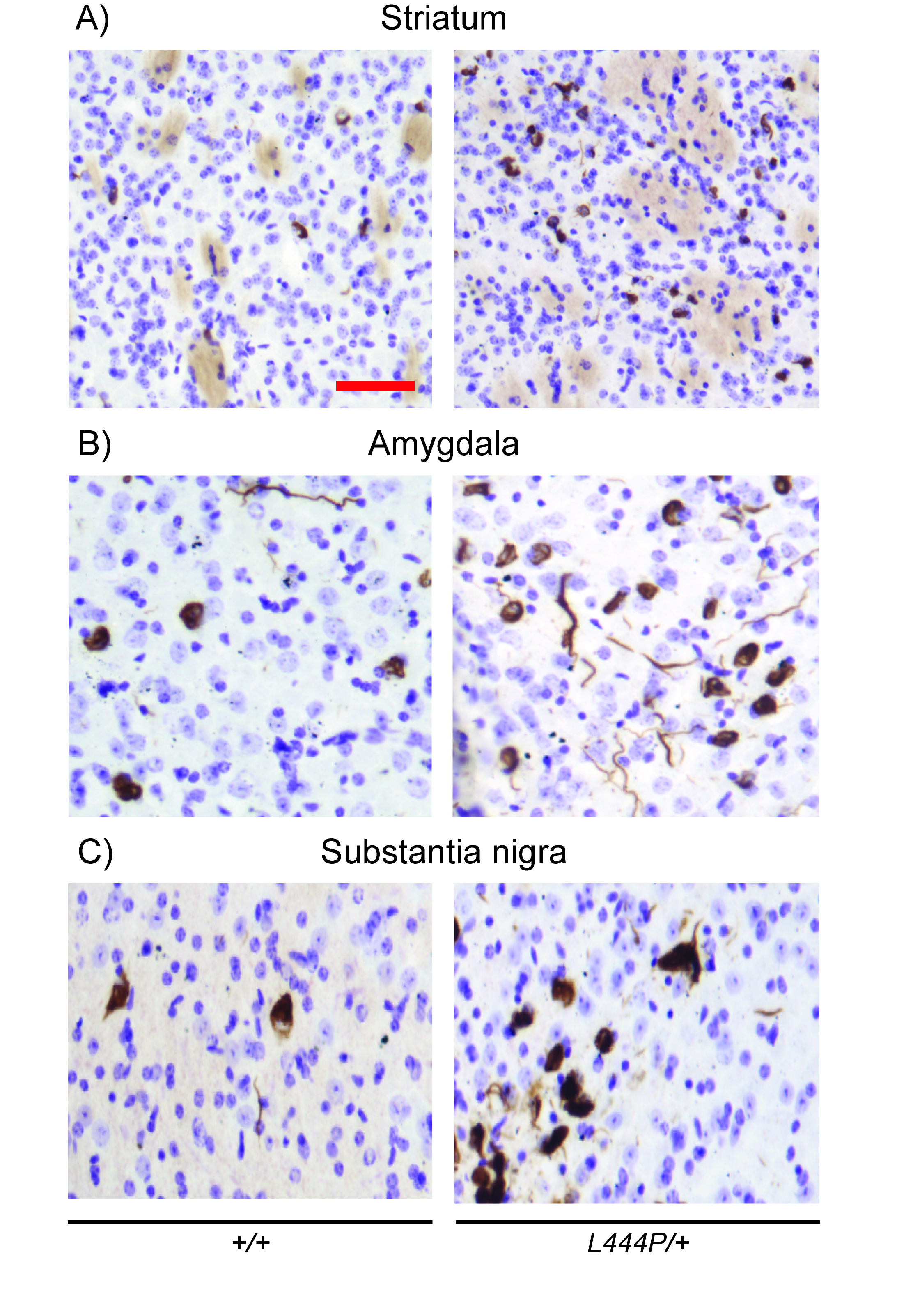

Supplement: S4 Fig — (A) Increased p-αSYN pathology in the striatal tissue at the level of 0.6mm anterior to the injection site (+0.8mm from the bregma) in the ipsilateral and contralateral hemispheres of L444P/+ mice compared to their wild-type control littermates. (B) Increased p-αSYN pathology in the lateral amygdaloid nuclei at the level of 2.2mm posterior to the injection site (-2.0mm from the bregma) in the ipsilateral hemisphere of L444P/+ mice compared to their wild-type control littermates. (C) Increased p-αSYN pathology in substantia nigra pars compacta at the level of 3.7mm posterior to the injection site (-3.5mm from the bregma) in the ipsilateral hemisphere of L444P/+ mice compared to their wild-type control littermates. Scale bars = 50µm. Representative images shown. In total ten +/+ and four L444P/+ mice were analyzed. (TIF) [file pone.0238075.s004.tif]

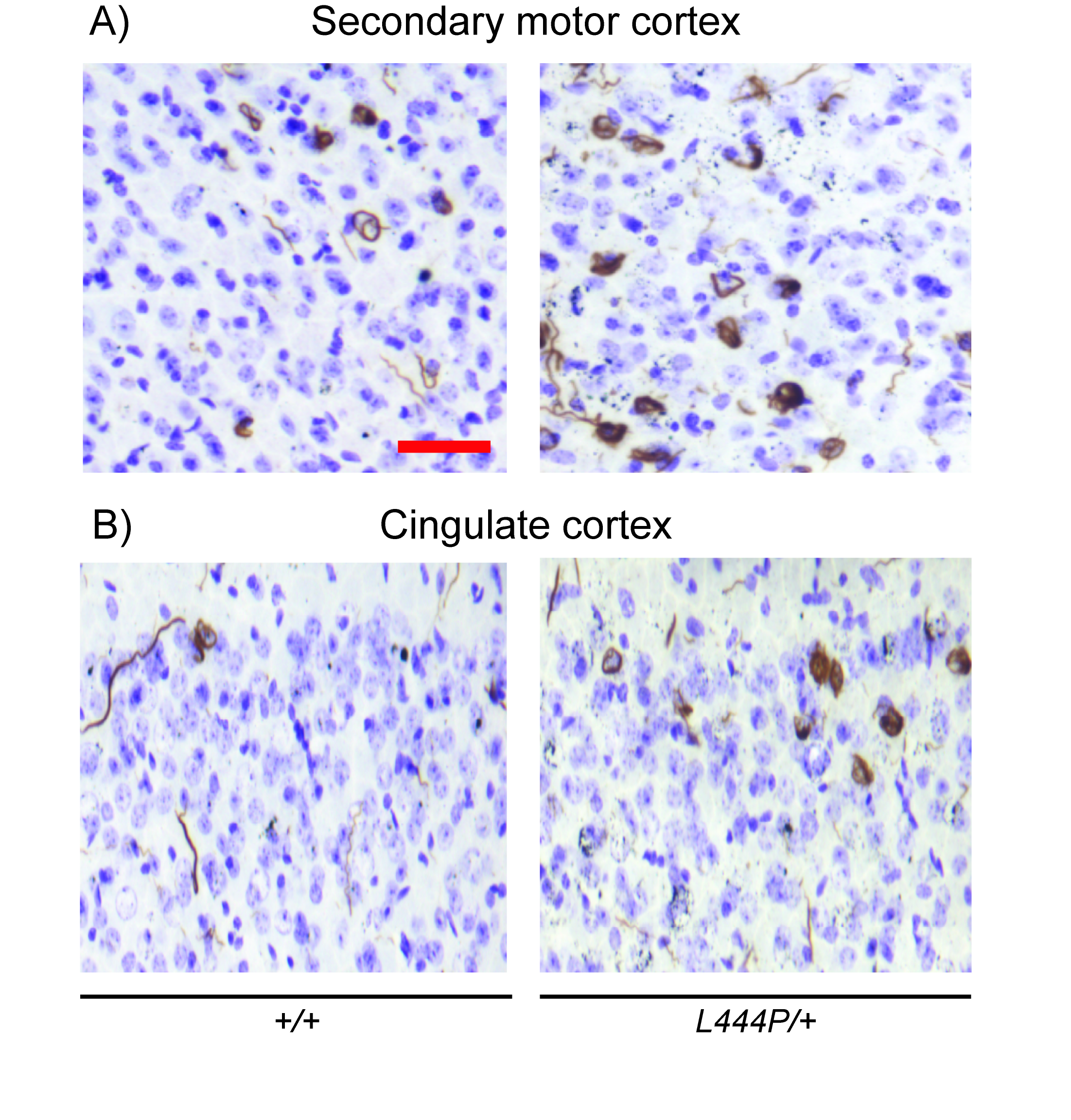

Supplement: S5 Fig — (A) Increased p-αSYN pathology in the cortical tissue at the level of 0.6mm anterior to the injection site (+0.8mm from the bregma) in layer 5 of the secondary motor cortex in the ipsilateral and in layer 2 of the secondary motor cortex in the contralateral hemisphere of L444P/+ mice compared to their wild-type control littermates. (B) Increased p-αSYN pathology in the cortical tissue at the level of 0.6mm anterior to the injection site (+0.8mm from the bregma) in the cingulate cortex in the ipsilateral and contralateral hemispheres of L444P/+ mice compared to their wild-type control littermates. Scale bars = 50µm. Representative images shown. Ten +/+ and four L444P/+ mice were analyzed in total. (TIF) [file pone.0238075.s005.tif]
