# Supplementary material for: L444P Gba1 mutation increases formation and spread of α-synuclein deposits in mice injected with mouse α-synuclein pre-formed fibrils
Source: PLoS One. 2020 Aug 24;15(8):e0238075. doi: 10.1371/journal.pone.0238075 (PMC7444808; doi:10.1371/journal.pone.0238075)
Supplement: Attachment — Submitted filename: Response to Reviewers.docx [file pone.0238075.s006.docx]

**We thank both the Editor and Reviewer for their comments.**

**Answers to both the Editor and Reviewer:**

**1. Why the relatively small number of L444P/+ animals that were analysed?**

We used a relatively small number of L444P/+ mice based on previous publications concerning pre-formed fibril injections and our own published data.

Luk, KC, Kehm V, Carroll J, Zhang B, O’Brien P, Trojanowski JQ, et al. Pathological α-synuclein transmission initiates Parkinson’s-like neurodegeneration in nontransgenic mice. Science. 2012a 338(6109):949-53.

In this paper, 3–7 animals were examined per group.

Luk, KC, Kehm VM, Zhang B, O'Brien P, Trojanowski JQ, Lee VM. Intracerebral inoculation of pathological α-synuclein initiates a rapidly progressive neurodegenerative α-synucleinopathy in mice. J Exp Med. 2012b 209(5):975-86.

In this paper, 3–12 animals were examined per group.

Migdalska-Richards AM, Wegrzynowicz M, Rusconi R, Deangeli G, Di Monte DA, Spillantini MG, et al. The L444P Gba1 mutation enhances α-synuclein induced loss of nigral dopaminergic neurons in mice. Brain. 2017 140:2706-2721.

In this paper, 3–6 animals were examined per group.

Based on the number of mice used in each of these 3 publications (please see above) and that with careful measurements, the number was (and is) expected to produce a statistically significant result. Also, as our funders (PUK) and institution (UCL) are committed to the 3Rs, the minimum number of animals was entered into the design.

**Answers to the Editor:**

**1. The data in Figure 2D and E should be presented as scatter plots so that the reader can visualize the variability in the data.**

The figures were changed accordingly.

**2. On page 8, line 182 the authors state that "the number of p-a-Syn deposits was elevated in the striatum of L444P/+ mice compared to WT littermates, but this was not statistically significant". These two phrases are mutually exclusive. If the statistics do not confirm that the level of deposits was elevated, then one cannot make the statement as written.**

The statement was changed to: “There was a trend for an increase in p-αSYN deposits in the striatum of L444P/+ mice compared to WT littermates, but this was not statistically significant.”

**3. Because you have unequal sample sizes, it is necessary to do a post hoc test of validity. If the variances in the data sets are similar, then you can use Tukey-Kramer or Fisher's test among other possibilities. If the variances in the data are not equal, then there are other post hoc tests that must be used.**

Typically, a post hoc test of validity is applied when ANOVA test is used to calculate a statistical significance. However, post hoc tests are not commonly used when t-Test is used to calculate a statistical significance. t-Test is commonly used to compare statistical changes between two groups, while ANOVA is typically to compare statistical changes among more than two groups. As we only had two groups, we chose t-Test for our analysis. We checked the equality of variances using Levene's test and we did not find any statistically significant changes between any analysed groups (p=0.304 for cortex and p=0.884 for striatum). We checked the data normality using Shapiro-Wilk test and found the normal distribution in all analysed groups (p=0.579 for WT/cortex, p=0.716 for L444P/cortex, p=0.052 for WT/striatum and p=0.959 for L444P/striatum). Thus, after confirming the normal distribution and the equality of variances, we could compare all the groups.

**4. With the journals making it relatively painless to provide supplementary data, it would be useful to show some images of the absence of pathology in the controls as supplemental data. Similarly, with an N of only 4, more images of pathology could be provided as supplemental figures.**

The Supplementary Figure 3 was generated to show the absence of p-αSYN pathology in PBS-injected control mice. The Supplementary Figure 4 and 5 were generated to provide additional images showing the presence of p-αSYN pathology in αSYN-PFF-injected L444P/+ mice.

**5. The resolution of the supplemental images is relatively low (even after downloading the better quality image).**

The better-quality images were generated.

**Answers to the Reviewer:**

**1. Only one antibody to pSer129 was used for immunostaining. For proper scientific validation and rigor, the findings should be confirmed with at least one addition antibody, preferably not to pSer129.**

We used a well validated antibody for pSer129 (see publications above), and we are unclear as to why another antibody not to pSer129 would have been of value.

**2. In Figure 2E, the quantification of the motor and cingulate cortices were combined. These should be separate.**

In the mouse, the cingulate and motor cortices are adjacent and the border between them often indistinct, making separate counting inappropriate. Therefore, for the purpose of illustrating pathology (see Figure 2) we selected representative sections taken from the periphery of the respective areas away from their border. However, for the purpose of quantitation we combined the two adjacent areas of cingulate and motor cortices to avoid mis-appropriation of data between the two.
